# Dual vortex breakdown in a two-fluid whirlpool

**DOI:** 10.1038/s41598-021-02514-6

**Published:** 2021-11-29

**Authors:** Sergey G. Skripkin, Bulat R. Sharifullin, Igor V. Naumov, Vladimir N. Shtern

**Affiliations:** 1 Kutateladze Institute of Thermophysics SB RAS, Novosibirsk, Russia 630090; 2 Novosibirsk State University, Pirogova Street. 2, Novosibirsk, Russia 630090; 3 Shtern Research and Consulting, Houston, TX 77096 USA

**Keywords:** Fluid dynamics, Techniques and instrumentation

## Abstract

Looking for an optimal flow shape for culture growth in vortex bioreactors, an intriguing and impressive structure has been observed that mimics the strong swirling flows in the atmosphere (tornado) and ocean (waterspout). To better understand the flow nature and topology, this experimental study explores the development of vortex breakdown (VB) in a lab-scale swirling flow of two immiscible fluids filling a vertical cylindrical container. The rotating bottom disk drives the circulation of both fluids while the sidewall is stationary. The container can be either sealed with the still top disk (SC) or open (OC). As the rotation strength (Re) increases, a new circulation cell occurs in each fluid—the dual VB. In case SC, VB first emerges in the lower fluid at Re = 475 and then in the upper fluid at Re = 746. In case OC, VB first emerges in the upper fluid at Re = 524 and then in the lower fluid at Re = 538. The flow remains steady and axisymmetric with the interface and the free surface being just slightly deformed in the studied range of Re. Such two-VB swirling flows can provide efficient mixing in aerial or two-fluid bioreactors.

## Introduction

A core of a vortex can abruptly expand, and its axial velocity can reverse. This phenomenon is referred to as vortex breakdown (VB). After the pioneer publication^1^ more than thousand papers have studied and discussed VB. This lasting interest from researchers is due to important applications and apparently enigmatic nature of VB. The VB is recognized to influence aircraft wings' lift and drag forces, flame stabilization in combustion chambers, mixing and mass transfer in chemical and biological reactors, and weakens tornadoes^2^.

Starting with works by Vogel^3^ and Escudier^4^, many fundamental studies of VB have been performed using flow in a sealed cylindrical container whose one end-disk rotates while all other walls are stationary. Such a flow is free of unpredictable ambient disturbances and has well-defined boundary conditions and control parameters. These favorable features allow for a meaningful comparison between precise experimental^4^ and numerical^5^ results that help understand the VB physics. In VB studies before 2015, only one fluid filled the container.

The development of aerial vortex bioreactors^6,7^ led to interest in two-fluid or multiphase flows. A propeller drives an air flow, which in turn drives a water flow. The air flow transports oxygen, required for tissue growth to the interface. The oxygen diffuses through the interface and dissolves in water. The meridional circulation of water enhances the mixing of the dissolved oxygen with other ingredients. Thus, the aerial vortex bioreactor provides the gentle, fine, and nonintrusive mixing of ingredients required for the efficient growth of tissue cultures.

A useful model of an aerial vortex bioreactor is a sealed vertical cylindrical container filled with two immiscible fluids having remarkably different density and viscosity. For instance, the air–water flow can be driven by the rotating lid while the other walls of the container are stationary. The simple geometry and well-defined boundary conditions are convenient for detailed experimental and numerical studies and can aid understanding some intriguing features of two-fluid swirling flows. One of these striking features is the development of dual VB.

Tsai et al. first observed^8^ VB in the upper fluid in a water/soybean–oil flow. Initially, the experimental studies of two-fluid flows used only photo and visual observations^8,9^. Numerical simulations revealed that a few changes in the flow topology occur in the lower fluid before the VB occurrence^10^ and significant deformation of the interface^11^ observed in the experiments^8,9^.

It is obvious that the transition from one-fluid to multi-fluid flows significantly complicates hydrodynamics, but two-fluid flows have a wide range of technical applications, mainly related to heat and mass transfer at the interface between different fluids. The study of immiscible-multi-fluid flows attracts considerable interest due to a number of technical problems arising in oil production and associated with the transport of a mixture of oil and water over long distances through pipes^12^, considering different velocities and phase ratios. Another set of problems, also related to oil production are flows in flat mini-channels, which simulates oil–water motions in a porous medium^13,14^.

In our experimental study, the interaction between two liquids is controlled by the centrifugal force. This way has applications in bioreactors, providing smooth and fine mixing of ingredients that is beneficial for the growth of biological culture. In addition, the use of two immiscible liquids, having different viscosities and densities, makes it possible to significantly expand the range of realizable velocities with a stable and pronounced interface.

Measuring two-fluid swirling flows is a challenge because the maximal velocity of the meridional motion is drastically smaller than the maximal swirl velocity, especially in the fluid remote from the rotating disk. In the first application^15^ of the particle-image-velocimetry (PIV), the experimental and numerical results agreed in terms of velocity distribution, including the VB appearance^10^ in the upper fluid (oil) contacting the rotating disk. However, velocity could not be measured in the lower fluid (water), because the velocity magnitude was too small to be well detected.

Naumov et al. overcame^16^ this limitation by replacing water with a water–glycerin solution whose viscosity was close to that of oil. This modification and the averaging of 200 PIV images (to increase the signal–to–noise ratio) allowed them to measure velocity and experimentally to identify flow patterns in the lower fluid as well. This helped reveal a paradoxical feature of two-fluid swirling flows—a discontinuity in radial velocity (slip) at the interface caused by the centrifugal force and a jump in density.

The one-fluid open flow was studied both numerically^17^ and experimentally^18^. Our work extends the experimental study of the VB development to the two-fluid flows. For comparison, we first consider the dual VB development in the sealed container, and then in the open one. Such approach helps highlight the important differences in the VB scenarios in these two cases.

The slip was confirmed in the further studies of VB in two-fluid flows^19–22^. The discovery of slip means that all numerical results, which were obtained under the continuity condition for all components of velocity at the interface, must be revised. As the slip makes problematic numerical simulations, importance enhances for further improvement of the experimental technique.

This study does advance the PIV experimental technique (Sect. 2) to better examine the VB development in the sealed (Sect. 3) and open (Sect. 4) containers. The obtained results are discussed (Sect. 5) and summarized.

## Experimental setup and measurement equipment

**Figure 1:**
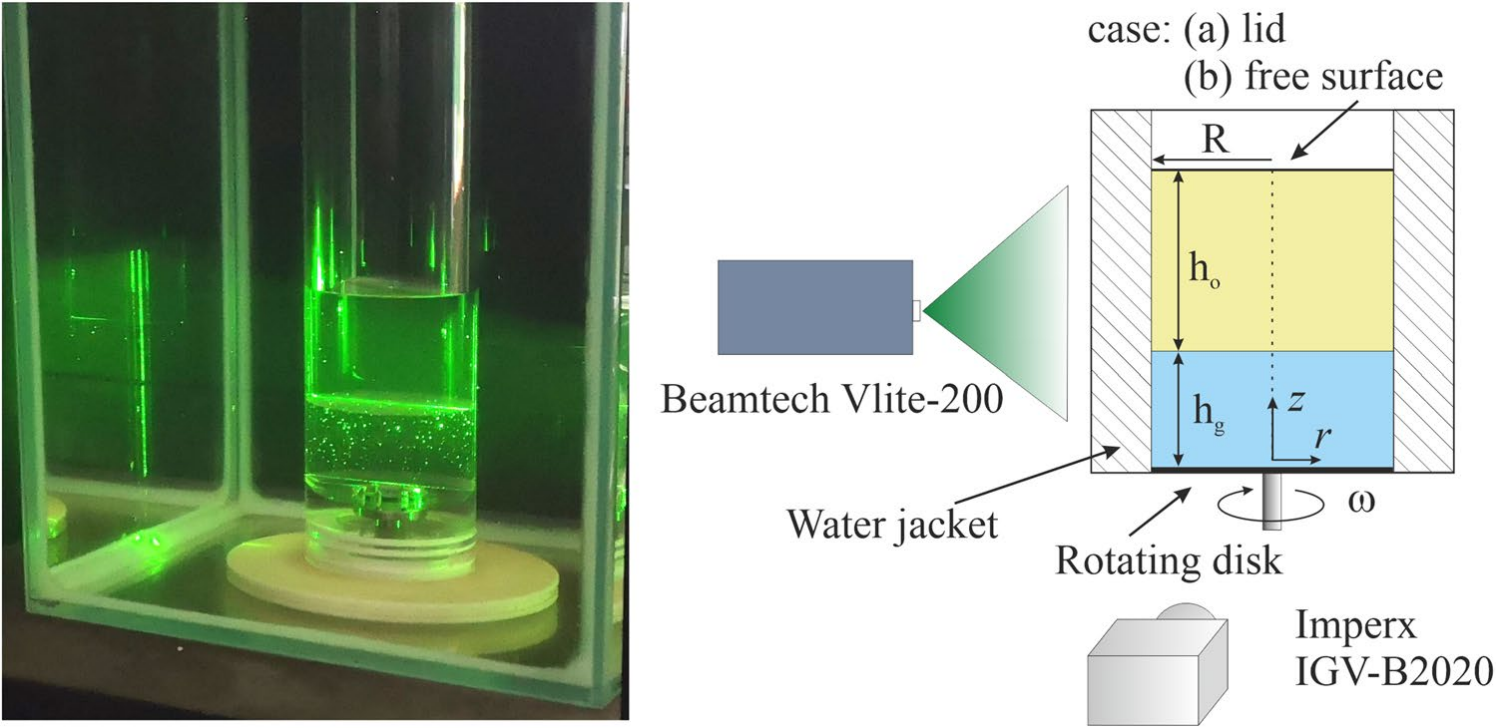
Photo of set-up (left), experimental setup scheme (right), *R* = 73 mm, *h* = 2.5*R*, *h*_g_ = *R*, and *h*_o_ = 1.5*R*.

Figure 1 demonstrates a photo of the experimental setup and a schematic view of the measurement system. The flow rotation is provided via the bottom disk of radius *R,* which rotates with angular velocity ω. The entire cylindrical container was placed inside a rectangular transparent box made of high-quality glass and filled with water to minimize thermal changes and optical aberrations which take place due to the cylindrical surface. The upper fluid is polymethyl siloxane oil of density ρ_o_ = 920 kg/m^3^ and viscosity ν_o_ = 8·10^−6^ m^2^/s. The lower fluid (glycerol) is a 65% distilled water and 35% glycerol solution of density ρ_g_ = 1070 kg/m^3^ and kinematic viscosity ν_g_ = 70·10^−6^ m^2^/s. The flow is kept isothermal at 22 °C. The swirling motion of flow is characterized by the Reynolds number Re = ω*R*^2^/ν_g_. The harmonic control signal is generated using a PC sound card and passing through the low-frequency amplifier to match the level, is fed to the input of the stepper motor driver. Rotational frequency after multiplying by the gear ratio of the stepper motor is transmitted by frequency modulation to the stepper motor driver SMD-4.2PL, which controls the stepper motor rotation providing required Re number.

The height of the lower and upper layers was chosen in such a way as to obtain bubble vortex breakdown in both liquids even in the laminar regime; with an increase in the height of the liquid layers, more energy is required. Thus, for the given ratio of the viscosities of the lower and upper liquid, it was possible to obtain the simultaneous onset of a bubble breakdown far from the Re numbers corresponded to an unsteady flow.

Due to the remarkable difference in the fluid densities, the interface remained stable during the experiment and did not have significant deformations for Re values required for the formation of VB in both fluids. The exact configuration of liquids was considered earlier^23^, which was focused on another interesting phenomenon—the slip of the radial velocity component at the interface.

Since the bubble VB onset maps for two fluids are unknown, a new visualization technique was used to determine the boundaries of the realized regimes. The track visualization system implements the adaptive real-time track visualization technique developed and tested by the authors^21,24^. Digital filtering based on averaging with a sliding window and subtraction of the static background were used to increase the signal-to-noise ratio, making it possible to observe low-contrast movements of light-scattering particles in a wide dynamic range even in the presence of significant background illumination. Used CMOS camera MC023MG-SY Ximea (resolution—2.3 MP 1936 × 1216, framerate up to 165 fps, sensor—Sony IMX174 LLJ-C).

Visualization processing algorithm propose next steps:

1. Gathering images for background image calculation A_m_ (A_m_—8-bit 1936 × 1216 matrix of intensities).
2. In real-time, extraction of the background matrix of intensities A_m_ from each collected image A_i_.
3. The last N_b_ images from the camera are stored, N_b_ is a current number of images in a buffer.
4. The matrices of intensities in the buffer are summed, and the average result image A is calculated:
  $$$$$$$$$$$$$$$$$${\text{A}} = \frac{{\mathop \sum \nolimits_{{{\text{i}} = 1}}^{{{\text{N}}_{{\text{b}}} }} \left( {{\text{A}}_{{\text{i}}} - {\text{A}}_{{\text{m}}} } \right)}}{{{\text{N}}_{{\text{b}}} }}.$$
5. When a new image is transmitted from the camera, steps 2 to 4 are repeated.

The length of the tracks in the resulting visualization is influenced by the frame exposure time and the size of the buffer N_b_. The larger buffer size provides longer tracks. This system allows for a precise tune of the observed regimes in real-time to get information about the Re number at which PIV measurements are required.

The velocity fields in vertical cross-section were measured using the Particle Image Velocimetry (PIV). Polyamide seeding particles, of density 1030 kg/m^3^ and diameter around 10 μm for lower fluid (0 < z < h_g_), and polyethylene microspheres, of density 950 kg/m^3^ and diameter around 5 μm for upper fluid (h_g_ < z < h_o_), to minimize the buoyancy effects, were employed as seeding light-scattering tracers for the PIV measurements. For each regime, 500 images were accumulated to perform velocity field averaging that increases the signal-to-noise ratio. Since the velocities in the lower and upper liquid are significantly different, measurements for each of them were carried out separately in order to find a suitable time delay between frames.

This PIV system is well approved in our early works for velocity measurements in a cylindrical container^16,23^. It consists of a double-pulsed Nd:YAG Beamtech Vlite-200 laser with a repetition rate up to 10 Hz, pulse energy 200 mJ, a CCD-camera IMPERX IGV-B2020, 8 bits per pixel, 4Mp matrix, and Polis synchronizing processor. Two-dimensional velocity fields were calculated using the ActualFlow software. The laser sheet thickness in experiments was about 1 mm in the measurement area. The camera was placed on a special mount system at 800 mm from the laser sheet plane for the vertical section.

We calculated the velocity fields using the iterative cross-correlation algorithm (3 inner iterations) with a continuous window shift and deformation and 50% overlap of the interrogation windows. In order to have a relatively large dynamic range (the span between the maximal and minimal velocity values), the size of the interrogation window initially was 64 × 64 pixels. Time delays between the two images varied from 50 to 200 ms depending on Re and fluid type. The background image averaged over 500 accumulated images was subtracted to reduce the noise level. The sub-pixel interpolation of a cross-correlation peak was performed over three points, using a one-dimensional approximation by the Gaussian function. The obtained instantaneous velocity vector fields were validated with the two subsequent procedures: the peak validation with the threshold 1.8 and the adaptive median filter over 8 × 8 nodes. The resulting velocity fields spatial resolution was about one vector per 1.5 mm.

**Figure 2:**
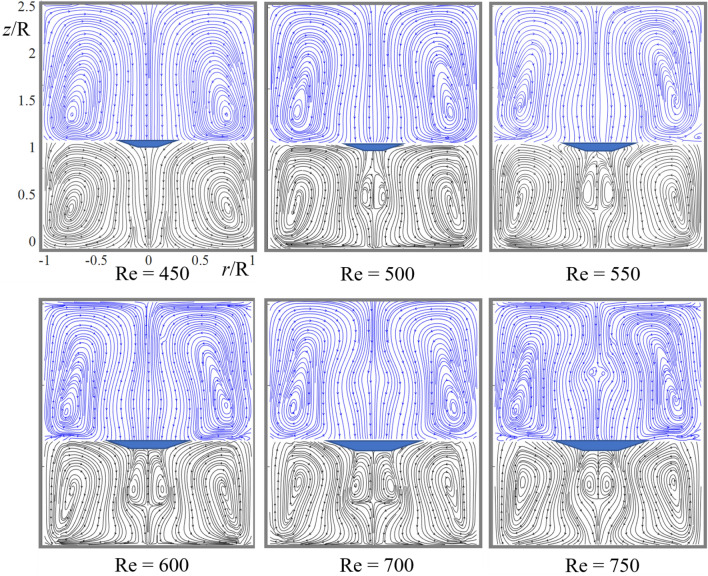
PIV streamline patterns show the development of dual vortex breakdown (VB) in the sealed container.

**Figure 3:**
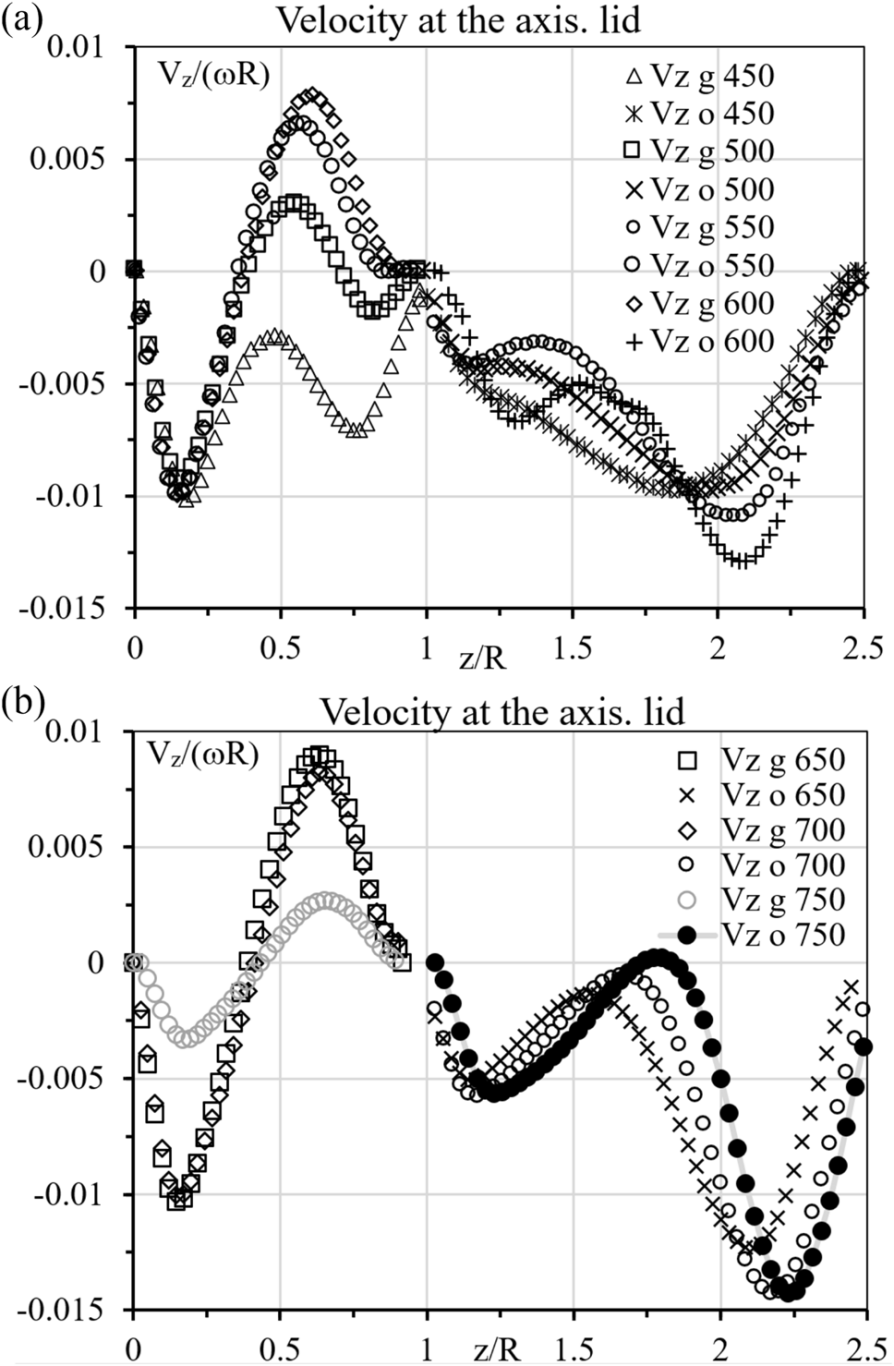
Symbols show the distribution of velocity at the axis in the glycerol (g) and oil (o). Numbers show values of Re, 450-600 (**a**), 650-750 (**b**). The ranges, V_z_ < 0 and V_z_ > 0, correspond to the centrifugal and anti-centrifugal circulations, respectively.

**Figure 4:**
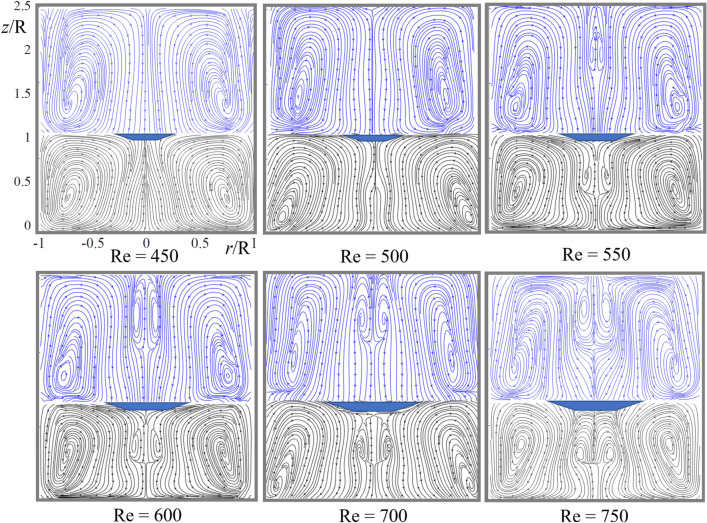
PIV streamline patterns show the development of dual vortex breakdown (VB) in the open container. As the Reynolds number (Re) increases, VB emerges first in the upper fluid and then in the fluid contacting the rotating bottom. (See Supplementary materials for track visualization of double bubble vortex breakdown at high Re.)

**Figure 5:**
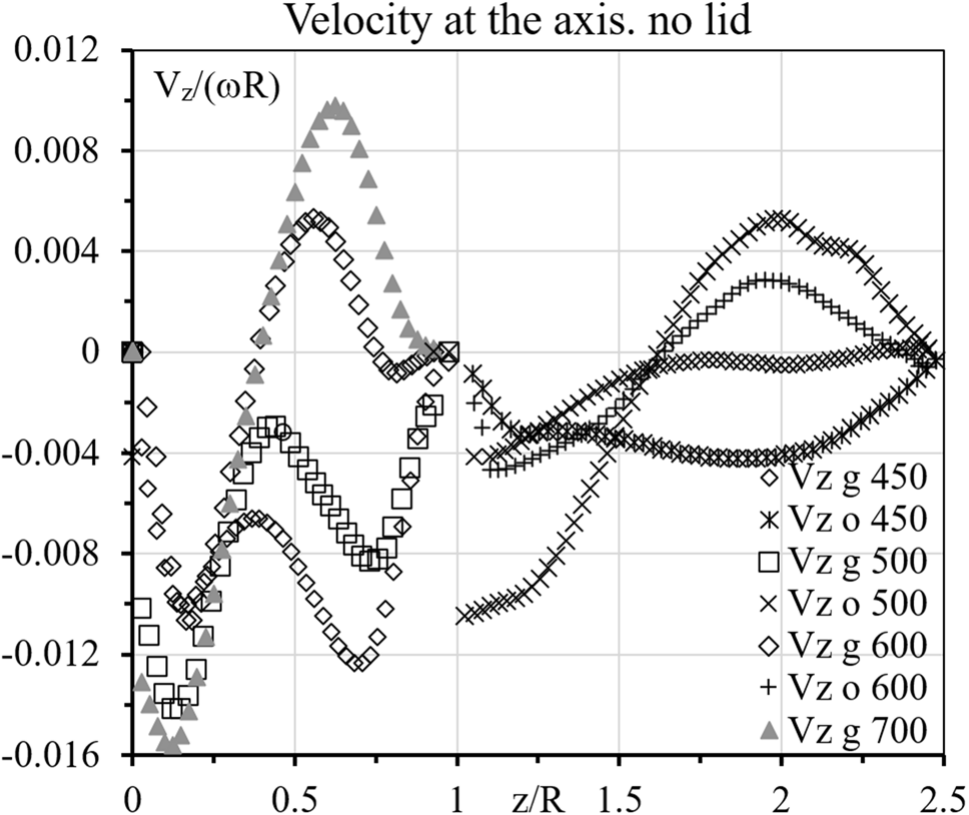
The notations are the same as those in Fig. 4, but the results are for the open container.

Thus, combining the traditional PIV method and the adaptive real-time track visualization technique allows us to record both statistical fields and velocity distributions (Figs. 2, 3, 4, 5) and the dynamic movement of recirculation zones in both fluids simultaneously (see Supplementary material).

## Dual VB in the sealed container

In this section, our experimental results are presented on the simultaneous bubble vortex breakdown onset in upper and lower liquids for sealed and open container flows. The flow patterns are provided via plotting of velocity streamlines in the vertical cross section for each Re values.

As Re increases starting from zero, three changes in the topology of the upper fluid occur^23^ resulting in the flow pattern shown in Fig. 2 at Re = 450 where both fluids centrifugally circulate. Accordingly, the velocity is negative (directed downward) at the axis in both glycerol and oil except at the interface and disks where V_z_ = 0; see Fig. 3 at Re = 450 where the velocity has the local maximum, V_z_/(ωR) = − 0.0029 at z/R = 0.49. At Re = 500, the maximum value is positive, V_z_/(ωR) = 0.0029 at z/R = 0.55. Interpolation yields that the maximum is zero at Re = Re_*l*_ = 475.

At this value of the Reynolds number, a new cell of anti-centrifugal circulation emerges – vortex breakdown occurs near the center of the lower fluid. Figure 2 shows that the cell is well developed at Re = 500. As Re further increases, the new cell expands mostly upward, touches the interface at Re = 600, radially widens along the interface at Re = 700, and starts to shrink at Re = 750.

As the Reynolds number (Re) increases, VB emerges first in the fluid, contacting the rotating bottom, and then in the upper fluid. The dark spot denotes the region between the fluids not resolved by PIV.

In the upper fluid (oil), the velocity at the axis remains negative in Fig. 3a, but the local maximum of V_z_(z) develops as Re increases. Figure 3b shows further growth of this maximum, which becomes positive at Re = 750 and vortex breakdown occurs in the upper fluid as Fig. 3 illustrates. Interpolation yields that the maximum is zero at Re = Re_u_ = 746. Thus, the dual vortex breakdown occurs for Re > 746. The difference in Re_*l*_ = 475 and Re_u_ = 746 is rather large. It becomes very small in the case where the top disk is absent, as shown next.

## Dual VB in the open container

At Re = 450 and 500, both fluids centrifugally circulate as Fig. 4 shows. The velocity is negative (directed downward) at the axis in both glycerol and oil except at the interface and disks where V_z_ = 0; see Fig. 5 at Re = 450 where the velocity has the local maximum. At Re = 500, V_z_/(ωR) = − 0.00295 at z/R = 0.439. At Re = 600, the maximum value is positive, V_z_/(ωR) = 0.00529 at z/R = 0.558. Interpolation yields that the maximum is zero at Re = Re_*l*_ = 538. The new cell is well developed at Re = 550 (Fig. 4). Therefore, in the open container case, vortex breakdown in the lower fluid occurs at the slightly larger value of Re (538) than that in the sealed-container case (475).

In the upper fluid (oil), the velocity at the axis also is negative at Re = 450 and 500 in Fig. 5, but the local maximum of V_z_(z) develops as Re increases. Figure 5 shows further growth of this maximum, which becomes positive at Re = 550 and vortex breakdown occurs in the upper fluid as Fig. 4 illustrates. Interpolation yields that the maximum is zero at Re = Re_u_ = 524 and z/R = 1.81. Therefore, in the open container case, vortex breakdown in the upper fluid occurs at the significantly smaller value of Re (524) that in the sealed container case (746) and even smaller than in the lower fluid (538) in the open-container case. Thus, the dual vortex breakdown occurs for Re > 538 nearly simultaneously in both fluids filling an open container.

## Discussion

To better understand the physics behind the obtained results, we use and test the swirl decay mechanism (SDM) of vortex breakdown development^2,25^. In a few words, SDM is the following. In high swirling flows, the azimuthal velocity is much larger than other velocity components in cylindrical coordinates. This feature allows for approximation reducing the radial momentum equation to the cyclostrophic balance, ∂p/∂r = ρv_θ_^2^/r, between the radial gradient of pressure and the centrifugal force. It follows from the balance that pressure at the vortex axis is smaller than at the vortex periphery and the pressure difference grows as the centrifugal force increases. If the swirl decays downstream, then pressure grows along the vortex axis and a local minimum of pressure develops. The lower pressure sucks ambient fluid, particularly along the axis, reducing and even somewhere reversing the axial velocity and thus producing recirculation regions (VB bubbles).

Consider whether SDM works in the current case. The rotating bottom disk feeds the attached fluid with angular momentum. The generated centrifugal force pushes the fluid from the axis to the periphery near the bottom, thus, driving meridional circulation of heavy fluid as shown at Re = 450 in Fig. 2. The fluid rises near the sidewall up to the interface where it turns toward the axis and goes downward as swirling jet, closing the loop.

The flow converging near the interface transports angular momentum close to the axis that according to the cyclostrophic balance, develops a local minimum of pressure near the interface-axis intersection. The low-pressure suction decelerates the near-axis jet and can reverse it forming a local counter-circulation (VB bubble), as Fig. 2 shows at Re = 500.

As Re increases, the bubble expands axially and moves up due to the suction, as Fig. 2 shows at Re = 550. Next, the bubble touches the interface and expands radially thus pushing away from the axis the converging motion, as Fig. 2 shows at Re = 600. The reduced convergence increases pressure at the axis and weakens the suction. The bubble shrinks and the circulation streamlines concentrate near the sidewall, as Fig. 2 shows at Re = 750.

Therefore, SDM well works here like in the one-fluid case, despite the different boundary conditions at the top: wall there and the interface here. A difference is in Re values at which VB emerges. Here this Re value is twice smaller than there due to less friction at the top. The SDM scenario of VB works in the lighter fluid as well, as Fig. 2 shows.

Paradoxical is the velocity jump at the interface: the heavy fluid moves to the axis while the light fluid moves away from the axis. as Fig. 2 shows. This counterflow emerges for Re < Re_t_ = 175^23^. For Re < Re_t_ (weak swirl) the upper fluid circulates clockwise driven by the convergent motion of the heavy fluid—the radial friction force.

The effect of rotation is opposite: the centrifugal force pushes fluid away from the axis. The circulation direction depends on what force dominates: radial friction or centrifugal. For Re > Re_t_, the centrifugal force dominates and first develops a small centrifugal circulation area near the upper fluid's interface-axis intersection. Then this area expands and occupies entire volume of the lighter fluid for Re > 250 resulting in the flow topology as shown in Fig. 2 at Re 450. The jump is well observed in Fig. 5 at Re = 450. It might be smoothed in a thin boundary layer near the interface, which our PIV measurements fail to resolve.

## Conclusion

Patterns of oil-glycerol immiscible two-phase rotating flow were experimentally studied. For the first time, a simultaneous dual bubble-like vortex breakdown has been observed in a container with a rotating bottom disk. The two cases with sealed (SC) and open (OC) container were explored. The case OC, with the free surface, in fact, corresponds to a flow of three fluids: glycerol, oil, and air.

In case SC, the shape and location of VB cells are similar for both glycerol and oil flows. In case OC, the shape of VB cell in the oil flow is thinner and axially more elongated than those in the glycerol flow. The VB develops in the oil flow at Re = 524 (OC) and 746 (SC). This difference is due to the upper still lid consumes the angular momentum, while the free surface does not. In the glycerol flow, the VB develops at Re = 538 (OC) and 475 (SC). This difference is relatively small because the lower-fluid flow is driven by the rotating bottom and weakly depends on the upper-fluid flow.

The large Re = 746 for the case SC is because the still disk consumes the angular momentum that must be compensated by addition supply from the rotating bottom disk, i.e., by larger Re than that (Re = 524) in an open container. The upper fluid motion is driven by the lower fluid motion at the interface, which serves as a liquid bottom for the upper fluid. In the open container the top surface is nearly stress-free that promotes the upper fluid circulation. We observed no visible effect of air on the liquid flow. It seems due occur to the thousand times difference in density.

The fact that in the glycerol, the VB develops at larger Re for the open container than that in sealed container indicates that the transfer of angular momentum from the lower to the upper fluid increase in the OC case. Since the OC case involves three fluids—glycerol, oil, and air—there is an open question: whether a triple VB occurring in each fluid can be observed?

The obtained results are important both for practical technological applications of multi-fluid flows (bioreactors) and for fundamental knowledge on the topology of swirling multi-component systems.

## Supplementary Information

Supplementary Information 1.

Supplementary Information 2.
