# Supplementary figures and images for: Dual vortex breakdown in a two-fluid whirlpool
Source: Sci Rep. 2021 Nov 29;11:23085. doi: 10.1038/s41598-021-02514-6 (PMC9022139; doi:10.1038/s41598-021-02514-6)

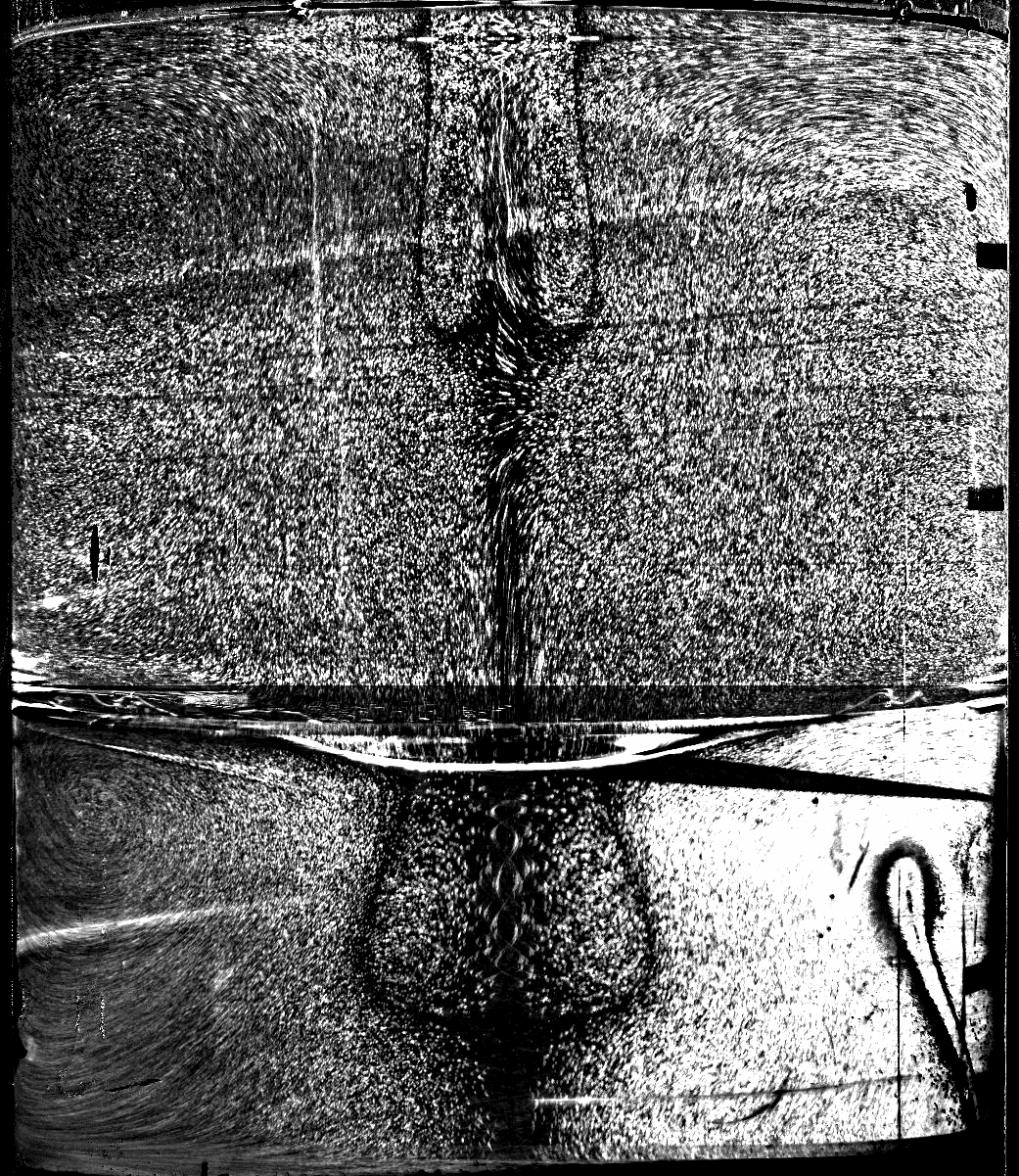

Supplement: Supplementary file 1 — Supplementary Information 1. [file 41598_2021_2514_MOESM1_ESM.gif]

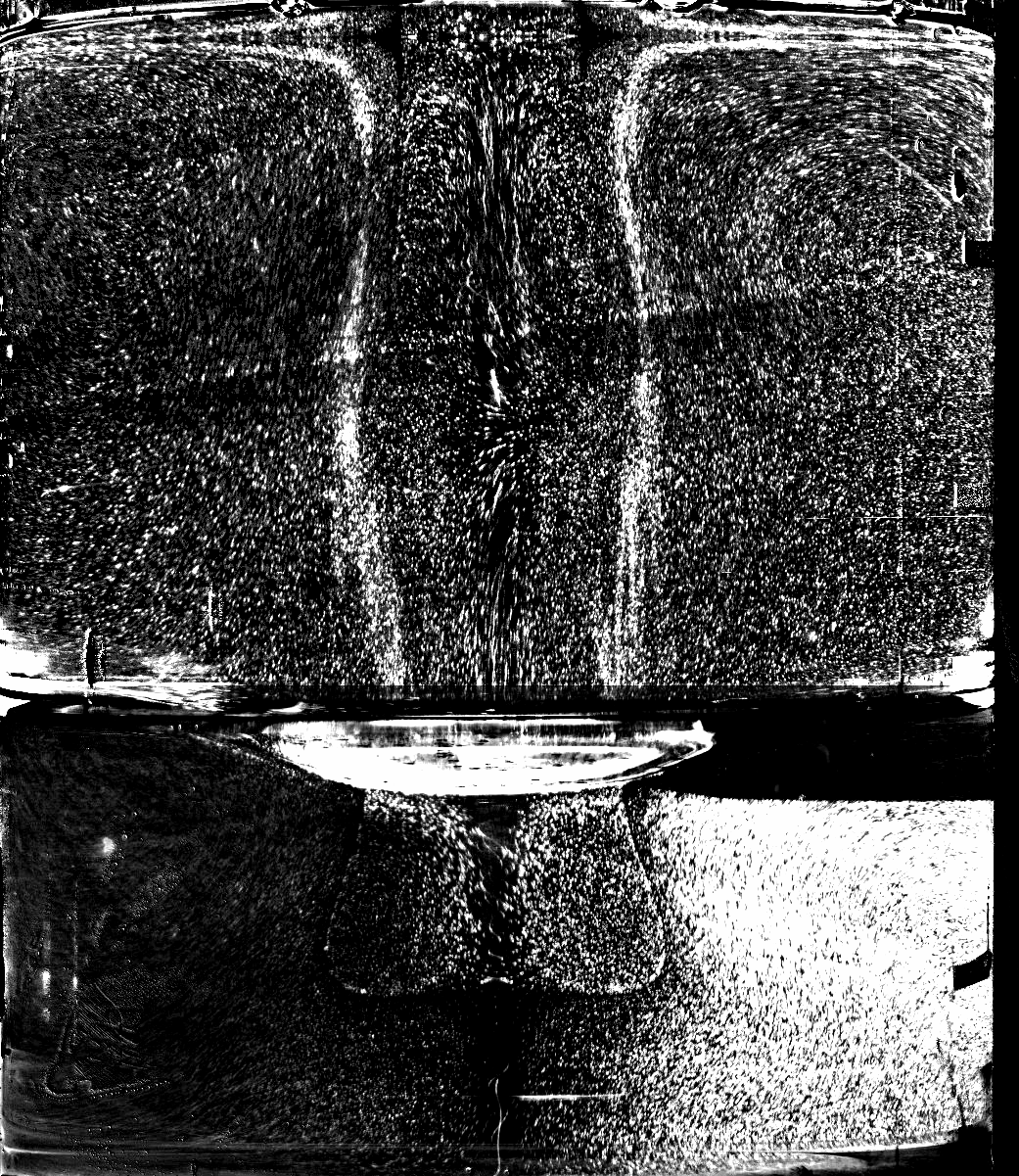

Supplement: Supplementary file 2 — Supplementary Information 2. [file 41598_2021_2514_MOESM2_ESM.gif]
